# The Power of Movement: How Exercise Influences Chemotherapy-Induced Peripheral Neuropathy

**DOI:** 10.3390/biomedicines13051103

**Published:** 2025-05-01

**Authors:** Joana Loureiro, José Tiago Costa-Pereira, Daniel H. Pozza, Isaura Tavares

**Affiliations:** 1 Unit of Experimental Biology, Department of Biomedicine, Faculty of Medicine, University of Porto, Alameda Prof. Hernâni Monteiro, 4200-319 Porto, Portugaljcostapereira@fcna.up.pt (J.T.C.-P.); dhpozza@med.up.pt (D.H.P.); 2 I3S—Institute of Investigation and Innovation in Health, University of Porto, Rua Alfredo Allen 208, 4200-135 Porto, Portugal; 3 Faculty of Nutrition and Food Sciences, University of Porto, Rua do Campo Alegre 823, 4150-180 Porto, Portugal

**Keywords:** chronic pain, neuropathic pain, pain modulation, antidepressants, serotonin, noradrenaline, review

## Abstract

As the number of cancer patients and survivors increases, we face a rising challenge: the long-term impact of the adverse effects of cancer treatment. One of the known adverse effects is chemotherapy-induced peripheral neuropathy (CIPN), which courses with pain complaints. The treatments of CIPN have reduced efficacy. The neurobiological causes of CIPN have been mainly ascribed to peripheral nerve damage, but recent studies show effects in the brain, namely in the descending pain modulatory systems. Physical exercise seems to be associated with better outcomes in CIPN patients, but the mechanisms underlying the effects have not been discussed, namely considering the recent results of the effects of CIPN in brain structures involved in pain modulation. In this critical review, we propose that the beneficial effects of exercise in CIPN also have central mechanisms, namely neuroinflammation and oxidative stress, as well as changes in the actions of neurotransmitters and neurotrophic factors, with a direct effect on optimizing the endogenous pain modulation, namely opioids, monoamines, and endocannabinoids. The effects are multifactorial, as mood improvement and the other psychological benefits of exercise should be considered. The emerging role of the microbiome, which is affected during CIPN, also needs to be considered. This review critically synthesizes the available literature to highlight how the neurobiological effects of physical exercise make it a promising strategy for managing CIPN, both from preventive and treatment perspectives.

## 1. Introduction

The growing incidence and prevalence of cancer are met by modern treatments that significantly extend survival, frequently shifting the classification of the disease into a chronic condition. Chemotherapy, a common cancer treatment, works by disrupting cell division and inducing apoptosis in both cancerous and healthy cells. Consequently, chemotherapy has adverse effects, some of which are well established, such as anemia, diarrhea, nausea, vomiting, immune depression, fatigue, hair loss, and infertility. Chemotherapy-induced peripheral neuropathy (CIPN) is also an important adverse effect of chemotherapy, which may lead to the reduction or even cessation of chemotherapy, causing clear compromise of cancer treatment and patient survival [1]. Furthermore, even after several years from the end of cancer treatment, some cancer-surviving patients continue to suffer from CIPN, with impact on their quality of life [2]. The chemotherapy agents known to induce CIPN include several drug classes, namely platinium-derived compounds (cisplatin, carboplatin and oxaliplatin), taxanes (paclitaxel and docetaxel), and vinka-alkaloids (vincristine) [1,2].

Patients with CIPN often present sensory symptoms such as tingling and numbness, and some complain of pain, namely spontaneous pain and increased sensitivity to innocuous (allodynia) or noxious (hyperalgesia) stimuli. Furthermore, since cancer is age-related, the patients with CIPN frequently face the sum of the consequences of aging, such as the risk of falls, as motor nerves are also lesioned during chemotherapy [3].

Physical exercise has general beneficial effects on health. According to several recommendations, it is proposed that cancer patients could benefit from doing at least 150 to 300 min of moderate-intensity aerobic activity, 75 to 150 min of vigorous-intensity activity, or a combination of both, combined with muscle-strengthening exercises at least 2 days a week [3], functional balance training at least 3 days a week [4], and flexibility training 2 to 3 days a week [5]. An increase in physical activity is associated with a reduced risk of cancer recurrence [6] and cardiovascular disease [7]. This can decrease the rate of mortality from cancer [8]. Moreover, the literature supports the safety of physical exercise during and after cancer treatment and further shows that supervised exercise interventions have more benefits than unsupervised programs [9,10]. The practice should be adapted to the patient’s abilities, starting with moderate amounts and undergoing incremental augmentation over the weeks. Besides the physical benefits, there is strong evidence regarding the benefits of physical exercise for anxiety and fatigue in cancer patients. In the specific case of CIPN, the current evidence is still insufficient for understanding the potential benefits of physical exercise in preventing and treating CIPN, which indicates that more research should be performed, with well-controlled interventions that have CIPN as the primary outcome [3].

Among its benefits, physical exercise prevents the loss of muscle mass; contributes to stability and gait, promoting balance control and a reduction in the number of falls; improves cardiovascular and metabolic fitness; and promotes pain reduction [11]. Therefore, it can be hypothesized that physical exercise has potential as a complementary therapeutic modality for CIPN, with exercise prescription adapted to individuals’ needs and abilities [12]. Nevertheless, it is important to discuss the neurobiological mechanisms that may subserve the possible improvements in CIPN prompted by physical exercise. In this critical review, we initially discuss the mechanisms of CIPN and of physical exercise to then set the grounds for a possible prescription of exercise to CIPN patients. This critical review aimed to (1) elucidate the underlying mechanisms of CIPN, with emphasis on recent results in the brain, namely in the areas involved in top-down modulation of sensory information and (2) propose a mechanistic hypothesis to explain how CIPN can be improved by physical exercise.

## 2. CIPN: Etiology, Prevalence, Agents, and Impact

CIPN is the main neurological adverse effect of many cytostatic agents [11]. It is a dose-dependent complication that affects 60–80% of cancer survivors treated with chemotherapeutic agents [13]. CIPN may present itself acutely, lasting from hours to days after treatment, or persist a month after finishing chemotherapy in 58–78% of patients [14] and become a chronic condition [15]. For example, oxaliplatin-induced neuropathy occurs in 90% of patients and usually reverses within one week. In contrast, more than 50% of patients receiving a cumulative dose of paclitaxel above 250 mg/m^2^ complain of paresthesia or hyperalgesia [16]. In addition, there are other factors that can increase the incidence of CIPN, such as the co-administration of other chemotherapeutic agents, and pre-existing conditions that affect the peripheral nerves, namely neuropathies due to alcohol consumption, traumatic neuropathies, and diabetes, situations in which the structure and function of peripheral nerves are already affected [17].

Typically, CIPN begins with sensory and motor deficits such as paresthesia, hypersensitivity to thermal stimulation, hyperalgesia and weakness in the extremities (or the “gloves and socks pattern”), postural instability, and pain [1]. Regarding postural instability, CIPN compromises patients’ safety, as it increases the risk of falling threefold compared to the general population. Additionally, there are autonomic changes such as gastroparesis and cardiac, urogenital, and sexual dysfunction [13,14]. These limitations reduce the patients’ quality of life by interfering with activities of daily living, such as dressing, writing, or even walking [13]. The clinical manifestations of CIPN depend on the cytostatic agent involved. Some agents like cisplatin, oxaliplatin, and carboplatin cause sensory and painful symptoms, while others, such as vincristine, induce sensorimotor symptoms with or without autonomic affectation [18]. Symptoms vary in intensity and duration, ranging from acute, transient sensations to permanent and sometimes irreversible nerve damage. There can even be a paradoxical worsening of symptoms after chemotherapy cessation, and CIPN may be exacerbated or even reappear. Besides these specificities, and compared to other neuropathies, CIPN often presents with more rapidly progressing and severe symptoms [1].

The consequences of CIPN on peripheral nerves have a multifactorial origin, and there are different possible mechanisms [16]. These mechanisms will be explored in detail later in this review. Because the primary means of alleviating CIPN symptoms involves reducing or stopping chemotherapy, these symptoms can significantly hinder cancer treatment’s efficacy and lead to suboptimal outcomes [13,19]. Currently, no drugs are specifically approved for CIPN treatment. Among the available options, the antidepressant duloxetine has shown some efficacy in CIPN-related pain, although it does not alleviate other symptoms and is associated with adverse effects, including nausea, dry mouth, constipation, and diarrhea [20]. Drugs approved to treat other neuropathies are less effective in the context of CIPN [16]. Several factors predispose patients to CIPN, such as age, pre-existing neuropathy, smoking, impaired renal function with reduced creatinine clearance, exposure to other neurotoxic agents, and paraneoplastic antibodies. Single-nucleotide polymorphisms (SNPs) that have been identified in the genome are also associated with a higher risk of CIPN [1].

Although CIPN does not exclusively occur in the elderly population, special consideration should be given to this demographic. With aging populations and increasingly advanced healthcare, many countries face a growing need for urgent rehabilitation of elderly patients after cancer treatment. Due to their increased susceptibility to chemotherapy’s adverse effects and the frequent need for dose reductions due to comorbidities, the elderly patients present unique challenges in cancer treatment. In addition, CIPN causes abnormal somatosensory feedback, which alters the representation of body position and movement in the central nervous system (CNS). The above-mentioned threefold increased risk of falling in patients with CIPN is obviously even higher in elderly patients with CIPN due to the already increased risk factors, such as the reduction in proprioception and bone/muscle masses. Consequently, particularly within this population group, therapeutic approaches should incorporate strategies to address balance deficits, and technological interventions targeting improved visuomotor coordination and increased body awareness have been proposed for elderly patients with CIPN [21].

## 3. Mechanisms of CIPN

Several mechanisms have been proposed for the pathophysiology of CIPN, depending on the cytostatic agents used.

### 3.1. Toxicity Induced by Platinum-Derived Compounds

Oxaliplatin, cisplatin, and carboplatin are examples of commonly used platinum-derived compounds. Oxaliplatin is indicated for tumors of the digestive tract, while cisplatin and carboplatin are indicated for various types of cancer, including small-cell lung cancer, testicular, ovarian, brain, uterine, and bladder cancers.

**Figure 1:**
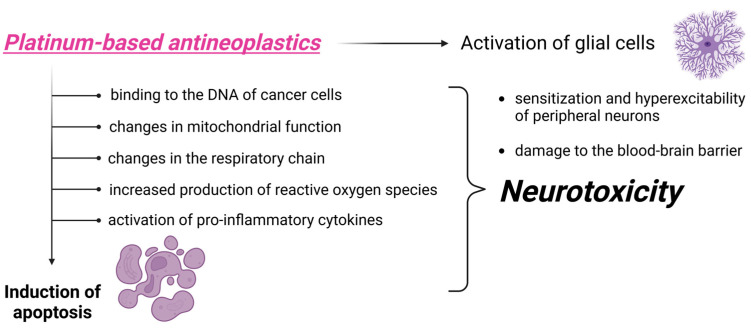
Mechanisms of CIPN induced by platinum-derived compounds. Created in https://BioRender.com (accessed on 18 February 2025).

The antineoplastic effects of these agents rely on inducing apoptosis through multiple mechanisms. They bind to cancer cell DNA, inhibiting replication and RNA transcription, which prevents cell division. Additionally, they alter mitochondrial function by forming irreversible mitochondrial DNA adducts (due to the lack of DNA repair in mitochondria), disrupting the respiratory chain, and increasing production of reactive oxygen species. These agents also activate the immune system by triggering pro-inflammatory cytokines, among other effects. It is assumed that these mechanisms cause neurotoxicity, due to the marked alterations that are caused both at the level of ion channels and membrane receptors, as well as intracellular organelles (examples of which include increased intracellular calcium concentration resulting in the activation of proteases and deregulated proteolysis, targeting the axon, and mutations in the genes that code for sodium channel proteins inducing pathology in the CNS and peripheral nervous system (PNS)). These agents activate glial cells, which attract and lead to the activation of immune cells and the release of pro-inflammatory cytokines, which lead to the sensitization and hyperexcitability of peripheral neurons. Ultimately, this neuroinflammatory cascade contributes to damaging the blood–brain barrier [1]. The mechanisms subserving platinum-induced neurotoxicity are summarized in Figure 1.

### 3.2. Toxicity Induced by Taxanes

Paclitaxel, docetaxel, and cabazitaxel are approved for the treatment of various cancer types, including ovarian cancer, breast cancer, non-small cell lung cancer and prostate cancer. CIPN symptoms are more intense with paclitaxel.

**Figure 2:**
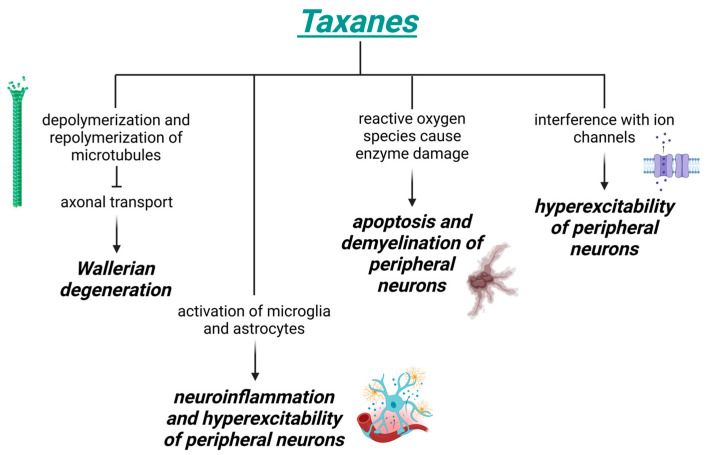
Mechanisms of CIPN induced by taxanes. Created in https://BioRender.com (accessed on 18 February 2025).

Taxanes disrupt the depolymerization and repolymerization of microtubules, preventing axonal transport and culminating in Wallerian degeneration, or the breakdown of distal segments of axons. Taxanes also interfere with the normal functioning of ion channels (sodium, potassium, and transient receptor potential (TRP) channels) and cause hyperexcitability of peripheral neurons [22,23,24,25,26,27,28]. In addition, taxane-induced mitochondrial damage increases the production of ROS, leading to enzymatic, protein, and lipid damage. This disrupts calcium homeostasis within neurons and promotes both apoptosis and demyelination of peripheral neurons [29,30,31]. Recent evidence shows that, besides the actions at the mitochondria, oxidative stress can also disrupt the function of another redox-sensitive organelle, namely the endoplasmic reticulum (ER), which may be relevant to CIPN [32]. Taxanes can also lead to the activation of microglia and astrocytes, which in turn recruit and and activate immune cells. This cascade results in the release of interleukins and chemokines, triggering a neuroinflammatory response that sensitizes nociceptors and exacerbates peripheral neurons hyperexcitability [22,33,34,35]. The mechanisms of taxanes that cause CIPN are shown in Figure 2.

### 3.3. Toxicity Induced by Vinca Alkaloids

The most neurotoxic agent from this group is vincristine, and it is used to treat various types of cancer, including leukemia, lymphoma, neuroblastoma, and Wilms tumor.

Vinca alkaloids do not easily cross the blood–brain barrier, but they can act on the cell bodies of peripheral neurons. These agents inhibit the conjugation of microtubules and promote their separation, destroying axonal transport. The attraction and activation of immune cells also lead to neuroinflammation [1]. Vinca alkaloids cause alterations in large neurons and those of the dorsal root ganglion (DRG), culminating in Wallerian degeneration [1].

**Figure 3:**
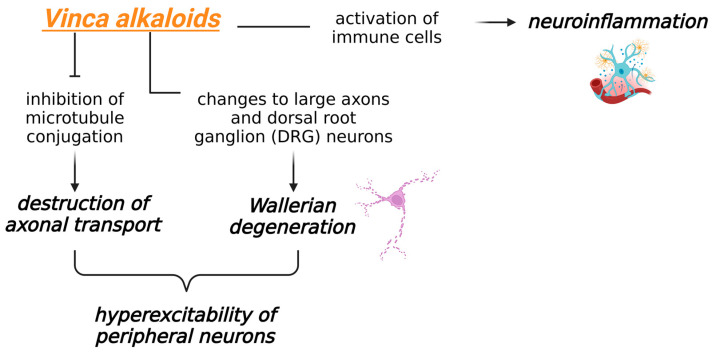
Mechanisms of CIPN induced by vinca alkaloids. Created in https://BioRender.com (accessed on 18 February 2025).

The mechanisms that cause CIPN are shown in Figure 3.

## 4. Central Effects of Chemotherapy-Induced Peripheral Neuropathy

All the above-mentioned changes induced by the three classes of chemotherapeutic agents in peripheral neurons affect their structure and function, disrupting stimulus processing and response, leading to CIPN symptoms. Brain hyperactivity—caused by factors such as reduced GABAergic inhibition, neuroinflammation, or overactivation of G protein-coupled receptor/mitogen-activated protein kinase (GPCR/MAPK) pathways—is linked to the severity of CIPN. Notably, hyperactivity is prominent in the insula, a key region for interoception [14,20].

Damage to peripheral nerves has central effects. CIPN causes alterations in the responses of the CNS to the arrival of peripheral input, namely increased ectopic signaling, decreased threshold of neuronal activation, and inappropriate connections that arise during nerve regeneration, contributing to neuropathic pain [36]. Through central processing, damage to the peripheral nerves leads to synaptic changes in the dorsal horn of the spinal cord, which facilitates the ascending transmission of the nociceptive information [20,36]. Furthermore, the accumulated evidence indicates that cytostatics also directly affect the brain, namely by inducing vascular alterations at the blood–brain barrier [37,38].

The effects in the brain impact the descending pain modulatory system, which includes neuronal circuits that balance facilitation and inhibition of nociceptive inputs, primarily using monoamines and opioids [39,40,41,42]. Alterations in the system compromise that balance and essentially favor the facilitation of the transmission of sensory inputs [43], which can contribute to the persistence of pain in CIPN, in a manner similar to other neuropathic pain conditions.

Neuroinflammation and neuroplastic changes also contribute to the pain associated with CIPN, through central sensitization and increased responses from the spinal and supraspinal neurons [44,45]. Neuroinflammation has traditionally been considered a peripheral mechanism. However, emerging evidence implicates this process in the brain and spinal cord, contributing to both the development and maintenance of CIPN. A study using rats has demonstrated that oxaliplatin increases levels of proinflammatory mediators and their receptors in the periaqueductal gray matter (PAG) [46]. Additionally, in animal studies, chemotherapy treatments increase proinflammatory cytokines, such as interleukin (IL) 1 beta, IL-6, IL-17 and tumor necrosis factor alpha (TNF-alpha) in the spinal cord [47,48,49]. Furthermore, studies have shown that cytostatic agents activate microglia and astrocytes in the spinal dorsal horn [48,50,51,52].

The brains of patients with CIPN who experience prolonged pain show changes, particularly in the limbic system [53,54]. Neuroimaging already shows that brain processing has a major impact on other conditions such as chronic low back pain or diabetic neuropathy [55,56,57]. One example is the study carried out by Nudelman et al. in 2016, in which brain scans were evaluated before and after chemotherapy in women with non-metastasized breast cancer, and it was concluded that increasing the severity of CIPN was related to an increase in perfusion of areas such as the superior frontal gyrus, cingulate gyrus, left middle gyrus, and medial frontal gyrus. After one month, severity was associated with increased perfusion of the left cingulate gyrus and left superior frontal gyrus. After a year, these relationships no longer existed [58]. Thus, it is suggested that CIPN is accompanied by structural and functional brain alterations [59]. A study using fMRI compared brain activity during painful heat stimulation in patients with CIPN and healthy controls [60]. The CIPN group showed greater activity in the left precuneus and lower activity in the right superior frontal gyrus upon nociceptive stimulation and increased activity in the left frontal operculum (near the insula). More recently, a prospective, multicenter cohort study used brain fMRI to study the development of pain complaints in CIPN patients nine months after chemotherapy. Increased activity in sensory, motor, attentional, and affective brain regions in response to punctate stimulation was demonstrated in CIPN patients with pain complaints [61]. Furthermore, the patients presented alterations in the activation of the PAG, an area that plays a central role in the descending modulation of pain, namely in conveying pain modulatory influences from higher brain centers, accounting for top-down pain modulation [10,11,12]. Depending on factors such as the emotional and cognitive conditions, a bidirectional system (inhibition/facilitation), controlling nociceptive transmission from the spinal cord, is triggered through multiple areas of the brain including the prefrontal cortex, anterior cingulate cortex, amygdala, and hypothalamus [62,63,64,65,66,67,68]. Besides higher brain centers, the PAG is also connected to other brainstem regions, such as the noradrenergic locus coeruleus (LC), the serotoninergic rostral ventromedial medulla (RVM), and areas of the medullary reticular formation, namely the dorsal reticular nucleus, an area involved in the facilitation of nociceptive transmission [39]. A recent study in animal models showed paclitaxel-induced neuroplastic changes in pain-related brain regions during CIPN. Increased neuronal activation was observed in the hypothalamus and PAG, along with shifting prefrontal cortex and hypothalamus metabolism, suggesting neuroplasticity and glial involvement [45].

Studies in animal models have shown increased activation of serotonin (5-HT) neurons may be due to activation of the p38MAPK pathway in the RVM which is activated in the CIPN [69], presenting excitatory projections of the PAG neurons, proposed to activate the neurons of the RVM and promote the facilitation of the descending pain pathway [70,71]. In the case of paclitaxel-induced neuropathy, 5-HT levels are increased in the spinal cord [71] but are decreased in treatment with oxaliplatin and cisplatin [72,73]. Thus, the involvement of 5-HT and the facilitation of the descending pathway must depend on the agent.

Modulation by the descending noradrenergic pathway is also affected by the neuroplastic changes involved in chronic pain [74,75]. The noradrenaline (NA) released into the spinal cord comes from the LC and, by activating the alpha-2A adrenergic receptor in the spinal cord, promotes analgesia by inhibiting nociceptive transmission in the dorsal horn of the spinal cord [76]. Indeed, paclitaxel-treated rats showed an adaptative enhancement of descending noradrenergic inhibition and augmented alpha-2A adrenergic receptor-mediated antinociception in the spinal dorsal horn [77]. Understanding the brain’s role in the pathophysiology of CIPN may pave the way for personalized strategies to manage and prevent this condition more effectively.

## 5. Exercise as a Complementary Approach to the Prevention and Treatment of CIPN

A holistic approach to cancer patients is important, and all findings that highlight potential benefits for the patient’s quality of life cannot be dismissed. Given the limited pharmacological options for CIPN, it is relevant to consider other complementary approaches to improve patients’ quality of life. Recent research focuses on modifiable behaviors, like physical activity, along with sleep. Patients associate insufficient exercise with increased pain, with inactivity further contributing to a cycle of pain and muscle weakness. CIPN severity is linked to fatigue and psychological distress, such as anxiety and depression, in a bidirectional relationship, where symptoms and distress reinforce each other [78]. Therefore, CIPN is a risk factor for depression and anxiety [79]. The relationship with sleep is bidirectional, since it is well established that a bad quality of sleep, in terms of duration and/or quality, worsens pain complaints [80].

There is evidence of a reduction in recurrence and mortality with the practice of physical exercise before and after cancer diagnosis [81]. It has been proven that regular physical exercise is beneficial for insulin sensitivity, cardiopulmonary and immune function, bone loss, body composition, depression, sleep disturbances, and quality of life in cancer survivors’ rehabilitation. The type of exercise implemented depends on the adverse effects of cancer treatment, namely CIPN, as well as the characteristics of the patient and the timing of the intervention [81].

Physical exercise seems to alter the processing of bodily sensations and trigger a neuroplastic phenomenon, independent of peripheral mechanisms, which relieves neuropathic pain [20,82], restoring normal inhibitory tone [83].

Greater physical activity is associated with a decreased likelihood of experiencing neuropathic pain when compared to lower levels of activity [84]. For those with CIPN, physical activity can be very beneficial as it improves balance and movement, reducing the risk of falls [85]. Additionally, not getting at least 150 min of moderate to vigorous exercise per week is linked to more severe symptoms, highlighting the importance of encouraging physical activity in chemotherapy patients [16]. Even when started after the beginning of chemotherapy, exercise can stabilize symptoms [86]. Based on the existing information, it can be suggested that the benefits of exercise for CIPN patients outweigh the risks, and therefore, it should be recommended [83].

In studies with rats, exercise after nerve damage was expected to reduce hyperalgesia. However, when serotonin [87] and dopamine [88] signals in the CNS were blocked, this exercise-induced pain reduction did not occur. This suggests that blocking serotonin and dopamine limits the beneficial effects of exercise in reducing pain after nerve damage, highlighting the importance of these neurotransmitters in the analgesic effects of exercise. In addition, the loss of inhibitory neurons in the dorsal horn, which produce gamma-aminobutyric acid (GABA) and modulate the afferent signaling of primary sensory neurons, is related to allodynia and hyperalgesia [36], and animal studies have suggested an association with the pathogenesis of neuropathic pain [89,90]. A preclinical study with animal models of CIPN—in this case mice—showed that voluntary exercise reduces mechanical and cold hypersensitivities, restores hippocampal cell proliferation, and increases cellular survival [91,92]. Moreover, it also mitigates mood dysregulation and motor impairment in animal models of CIPN, along with reduced anhedonia-like behavior and improved motor skill acquisition [91]. Chemotherapy decreases quality of life and is often accompanied by inactivity, as mentioned above, which worsens CIPN. The effects of treatments and their side effects force inactivity, creating a “cycle of inactivity” that leads to muscle weakness and lack of balance, causing even more inactivity. As a result, cancer patients and survivors are permanently at risk of the consequences of physical inactivity [93].

The potential benefits of physical exercise in the treatment of patients with CIPN have become a subject of increasing interest, thus justifying the rise in recent studies on this topic. The current body of evidence suggests that, in addition to being safe, exercise provides benefits even for patients undergoing palliative treatment, enhancing independence in activities of daily living by increasing strength, endurance, and balance [86,94]. The association between increased muscle volume and reduced symptoms of CIPN is noteworthy, and the efficacy of exercise appears to be pronounced in elderly individuals [20]. Furthermore, exercise has been demonstrated to facilitate nerve regeneration and is relevant to the recovery of neuromuscular function [95]. The normalization of aberrant connections in CIPN, particularly at the level of the structures of the interoceptive system, as visualized by neuroimaging, suggests that physical exercise plays a protective role in the structural brain alterations induced by CIPN [96].

Table 1 summarizes the main findings of randomized controlled trials (RCTs) and clinical trials aiming to evaluate the potential beneficial relationship between physical exercise and CIPN. The findings of these studies generally indicate symptomatic improvements in patients diagnosed with CIPN after being enrolled in a programme of prescription of physical exercise.

## 6. Mechanisms for Exercise Improvement in CIPN

Physical exercise can be a useful tool for patients with CIPN due to several neurobiological mechanisms that will be discussed below. To summarize, physical exercise has clear neurobiological effects on the somatosensory system that counteract the neurotoxicity of cytostatic drugs. Additionally, the psychological well-being associated with exercise is also an important factor to consider. An emergent factor to be considered is related to the putative actions of exercise in the gut–brain axis, which is affected during CIPN. Eight mechanisms can be considered, namely (1) neurotrophic factors; (2) anti-inflammation and antioxidation; (3) the opioid system; (4) the monoaminergic system (NA/5-HT); (5) the endocanabinnoid system; (6) human brain networks; (7) psychological well-being; and (8) the gut microbiome.

### 6.1. Effects on the Somatosensory System

Most of the studies on the effects of exercise in CIPN focus on the peripheral nervous system, proposing that exercise counteracts the direct and indirect damage caused by cytostatic drugs [14], but the underlying mechanisms are not yet fully understood [85]. As mentioned above, CIPN is linked to oxidative stress and neuroinflammation, leading to neurodegeneration and demyelination. Glia cells release pro-inflammatory mediators such as TNF-alpha, IL-1beta, IL-6 and IL-8, both through receptor-mediated pathways and indirectly through the activation and accumulation of macrophages. CIPN also affects neuronal function, leading to spontaneous activity and sensitization, which may account for hyperalgesia and allodynia [84,97,98]. Exercise may counteract those effects. In animals with traumatic nerve damage, treadmill exercise was shown to increase the expression of neurotrophic factors, namely glial cell line-derived neurotrophic factor (GDNF), brain-derived neurotrophic factor (BDNF) and insulin-like growth factor (IGF-1), which has been associated with axonal regeneration [99]. Despite the dual role of neurotrophic factors [100], the (1) neurotrophic hypothesis may be relevant for the beneficial effects of exercise in CIPN. Another possibility is the (2) anti-inflammatory and antioxidative effects of exercise. Muscle contraction is known to promote the release of anti-inflammatory cytokines, namely IL-10 and IL-1RA, and to reduce the levels of several oxidative stress markers [14].

Regarding the effects of exercise in pain, aerobic, resistance, and isometric exercise have been shown to have an analgesic effect in chronic pain [101,102]. An action in specific neurochemical systems has been shown namely in neurotransmitters that are present in several areas of the somatosensory system and play a role in pain modulation from the brain, with emphasis on opioids, monoamines (NA and 5-HT), and cannabinoids. The (3) opioidergic system includes opioid peptides (endorphins, enkephalins and dynorphins) binding primarily to mu, delta, and kappa opioid receptors (MOR, DOR, KOR), mediating analgesia in the CNS (brainstem, medial thalamus, medulla, hypothalamus and limbic system) [103] and PNS [104]. This pathway involves Ca2+ and K+ channel modulation, reducing neuronal excitability and neurotransmitter release [105]. Analgesia may result from excitatory connections between the PAG and the raphe nucleus magnus and release of opioids in these pain control areas [106]. Exercise increases plasma beta-endorphin levels proportionally to intensity [107], both in aerobic and resistance exercise [108]. Naloxone, an opioid receptor antagonist, reverses the exercise-induced analgesic effect and euphoria [103,109,110,111].

Additionally, the (4) noradrenergic system is activated during exercise via cardiovascular control and catecholamine release [112]. Catecholamines bind to alpha2-adrenergic receptors (A, B, C), modulating nociception. Receptors A and C inhibit cyclic adenosine monophosphate production, promoting K+ channel opening and Ca2+ channel closure, reducing excitability [113]. These receptors are found in the PAG, locus coeruleus, and DRG [114] and can activate the NO/cGMP pathway [115]. As to (4) 5-HT, exercise was shown to increase 5-HT levels in brain areas associated with pain control [116,117,118].

The (5) endocannabinoid system includes cannabinoid receptors type 1 and 2 (CB1 and CB2) in the CNS, particularly in pain-related areas like the PAG and spinal dorsal horn [119,120]. Activation by endocannabinoids reduces neuronal excitability and nociceptive transmission [121]. This system contributes to muscle vasodilation, euphoria, and bronchodilation during exercise [122], also involving the cerebellum and hypothalamus for motor control and thermoregulation [119,123,124]. Exercise increases endocannabinoid levels [124,125]. Cannabinoid receptor antagonists reverse exercise-induced analgesia, while anandamide reuptake inhibitors and endocannabinoid metabolism inhibitors potentiate it [126,127]. Analgesia involves the NO/cGMP pathway, with CB1/CB2 agonists reducing mechanical allodynia and thermal hyperalgesia through the NO/cGMP/K+ ATP pathway [128,129]. This system can also activate noradrenergic neurons [130].

Besides those neurochemical mechanisms, the involvement of NO should also be considered due to the correlation of its production during exercise and pain reduction [131]. NO increases post-exercise in plasma and cerebrospinal fluid, with higher expression in the dorsolateral and ventrolateral PAG, contributing to pain inhibition [132]. An interaction between NO release and the serotoninergic system should be considered, since NO stimulates 5-HT release [133]. Collectively, the results show that it is possible to conclude that there are several neurobiological mechanisms acting in the somatosensory system that account for the beneficial effects of exercise in CIPN.

### 6.2. Well-Being and Psychological Effects

Physical exercise is well known to be associated with mental health improvement [134], and group exercise programs, which foster a supportive and welcoming atmosphere, have shown psychological benefits by enhancing comfort and positive experiences, thereby improving adherence and outcomes [81]. Physical exercise also causes changes in functional neuroanatomical circuits such as the (6) salience network, executive control network, and default mode network, which may account for the well-being induced by exercise [135]. Interestingly, conditions such as anxiety and depression are associated with reduced volume in the hippocampus (part of the default mode network, which has been extensively studied for its responsiveness to exercise and its implication in the pathogenesis of depression) [136,137], amygdala, and anterior cingulate cortex (part of the salience network) [138]. These conditions are also linked to fewer connections between these different areas [139] and are characterized by deficits in cognitive flexibility and attention control [134].

Exercise also leads to changes in neural connections, which may help to explain some behavioral changes observed in marker studies. Exercise seems to be related to increased cognitive control and the functional connectivity of human brain networks [135,140,141]. There is evidence that exercise seems to increase connectivity in important neurocircuits [135]. In addition to neurotransmitters, (7) psychological well-being is also associated with the involvement of the hypothalamic–pituitary–adrenal axis, endorphins (which are associated with euphoric effects and may explain the feeling of well-being associated with exercise) and neurotrophic factors (associated with neuroprotection and regulation of mood disorders) [142].

Preliminary results from a recent randomized clinical trial demonstrated that home-based walking and resistance exercise may help mitigate CIPN symptoms and signs, while they increase default-mode network connectivity [96]. However, more neuroimaging studies with larger populations samples are needed to fully understand the neurobiological effects of exercise in the default-mode network circuitry of the brain.

### 6.3. Emerging Fields: The Gut Microbiome and Sex Differences

While clinical trials are lacking, preclinical and indirect clinical evidence suggests that exercise might positively influence (8) gut microbiota composition, leading to reduced inflammatory signaling and better neuroprotection in the context of CIPN. The gut microbiome influences immune and neuronal signaling, playing a crucial role in neuropathic pain [143]. Proinflammatory cytokines (e.g., TNF-alpha, IL-1 beta) and chemokines form a neuroimmune communication network, promoting neuroinflammation, peripheral and central sensitization, which contribute to neuropathic pain [144,145]. Interestingly, some Lactobacillus strains enhance anti-inflammatory mediator IL-10 production, suppressing neuropathic pain-related inflammation [146]. The alteration of gut microbiota affects microglial activation, indicating a microbiota-dependent regulatory mechanism. Moreover, metabolites from gut microbiota, like short-chain fatty acids (SCFAs) may impact microglial activation [147] and some bacterial strains, e.g., Lactobacillus and Clostridium, sporogenes produce metabolites, which limit astrocyte-related inflammation [146].

Emerging evidence shows a clear link between the gut microbiome and the establishment and maintenance of CIPN [148,149]. Indeed, an interesting connection between gut microbiota and CIPN has been reported, with reduced oxaliplatin-induced mechanical hypersensitivity observed in germ-free or antibiotic-treated mice [150]. Also, Ramakrishna et al., also using mice, found that the gut microbiota is essential in paclitaxel-induced neuropathic pain, since the cytostatic administration disrupts the microbiota–gut–brain axis communication, affecting CNS function [151].

Exercise training influences gut microbiota, as continued high-intensity exercise induces both taxonomic and metabolic changes in the microbiome [152]. A recent study has demonstrated that treadmill exercise improves memory in aged rats, reduces inflammatory markers, and enhances microbiota diversity [153]. Moreover, physical exercise appears to have a relevant antidepressant effect, potentially mediated by its ability to improve microbiota composition and diversity [154]. Based on this, it is plausible that physical exercise exerts a significant impact on the onset and progression of CIPN by reestablishing microbiome dysbiosis. However, the direct connection between exercise, the gut microbiome, and CIPN needs to be studied in future.

Another issue to be considered is the existence of possible differences in the effects of exercise in males and females suffering from CIPN. Sex differences in the response to physical exercise are well established and may be related to musculoskeletal differences and immune effects. As for the muscle system, men generally possess greater muscle mass and anaerobic power, largely due to their higher testosterone levels, and there are some suggestions of sex-specific mechanisms in CIPN muscle wasting [155]. As for immune effects, high-intensity exercise might temporarily impair immune responses to a greater degree in women, and this may have consequences in some types of cancers [156]. Research is necessary to understand the responses among different populations, namely in what concerns sex differences in the prescription of exercise to CIPN patients.

Table 2 summarizes the mechanisms that may underlie the effects of exercise in CIPN.

## 7. Conclusions and Future Perspectives

Building upon the mechanistic hypotheses explaining exercise’s positive impact on CIPN-related quality of life, this review underscores the need for further basic and clinical research into exercise interventions. Moreover, future investigations should also explore the potential benefits of other lifestyle modifications, such as targeted dietary supplementation, in managing CIPN. Combining nutritional interventions, such as probiotics, with exercise may be a promising strategy through which to restore gut microbiota balance and potentially alleviate the adverse effects of CIPN. By addressing the complex interactions between exercise, nutrition, and the gut microbiome, future research has the potential to produce clearer insights into how these interventions can be optimized for cancer patients undergoing chemotherapy.

Multidisciplinary teams are crucial for stratifying a patient’s risk of developing CIPN. To maximize engagement and leverage psychological benefits like belonging and motivation, patients should have diverse exercise options, ranging from professionally supervised programs to community-based and group activities. Furthermore, future research should investigate pre-morbid physiological factors and behaviors that might predict the onset of CIPN before chemotherapy begins. Personalized programs that motivate patients to change or improve their lifestyle will be the future approach to rehabilitation. The American Society of Clinical Oncology advocates for regular assessment of physical activity as a vital sign and encourages oncologists to discuss exercise with cancer patients. Supervised exercise tends to produce better outcomes, and while oncologists may not prescribe specific exercises, they should direct patients to appropriate professionals [3,100,157].

Therefore, physical exercise occupies a promising position as a possible complement to conventional treatment for CIPN, and the future of research should continue to mechanistically explain the benefits of exercise in CIPN and personalize exercise programs to patients, their disease, their routine, and their expectations. Questions of sex differences in exercise prescription in CIPN may also be important as an area of research in the future. Given the effects of physical exercise at both peripheral and central levels, it can be used as a tool to prevent the progression of CIPN or, once established, to reduce its severity, thereby improving the quality of life for these survivors. More robust RCTs are necessary, especially on recurrence and survival and on the use of physical activity in oncology rehabilitation and follow-up, and we should aim to better understand the role of physical exercise, particularly prior to chemotherapy, in preventing CIPN.

## Figures and Tables

**Table 1:** Overview of studies assessing exercise interventions in patients with CIPN (presented in chronological order).

Reference	Intervention	Methods	Outcomes	Statements from Authors
[94]	Conventional physiotherapy + supervised endurance (walking exercise in the hallway—6 min and stair walking exercise—2 min, 5 days a week) and strength training (abdominal exercise, a biceps curl exercise, and a triceps extension exercise for 3 sets, with patient-dependent number of repetitions, the other days) while the patient received three cycles of chemotherapy (breathing techniques or manual therapy)	(1) RCT; 46 lung cancer patients in stages IIIA/IIIB/IV who received palliative platinum-based chemotherapy were randomized into two groups: additional strength and endurance training program under the supervision of a licensed physiotherapist or only conventional physiotherapy; (2) After the intervention, they applied scales such as EORTC QLQ C-30/LC-13 questionnaire and Barthel Index	(1) Significant differences were detectable in the Barthel Index (independence in carrying out activities of daily living) in single scores of the EORTCQLQ C-30/LC-13 questionnaire (physical functioning, hemoptysis, pain in arms or shoulder, peripheral neuropathy, cognitive functioning), in the 6 min walk test, stair walking, strength capacity, and in the patient’s dyspnea perception during submaximal walking activities (Intervention Group > Control Group)	(1) “The training program has a positive impact on the patient’s independence in carrying out activities of daily living”; (2) “The training has a positive effect on the patient’s endurance and strength capacity”; (3) “This study demonstrated that even lung cancer patients receiving a palliative chemotherapy treatment should have enhanced physical activity intervention”
[86]	Eight-week supervised exercise program, including endurance, resistance and balance training (2×/week for 60 min)	(1) Two-armed, monocentric design. Metastasized colorectal cancer patients were allocated to an intervention group (*n* = 17) attending an exercise program or a waitlist control group (*n* = 13) which received written standard recommendations to obtain physical fitness; (2) All patients were assessed at baseline (t0) and after the intervention (t1) + follow up 4 weeks (t2)	(1) Neuropathic symptoms remained stable in the intervention group over time, while CIPN significantly worsened in the control group from t0 to t1 and t0 to t2; (2) The intervention group significantly improved in strength and balance function; (3) Changes in CIPN correlated with changes in balance	(1) “This study provides first evidence that a multimodal exercise program counteracts a worsening of CIPN and further improves balance and strength in a palliative setting with patients suffering from mCRC”
[20]	Six-week home-based exercise program (EXCAP: walking prescription, increasing the total number of steps walked daily by 5–20% each week + therapeutic band prescription, with varying levels of resistance)	(1) Secondary analysis of an RCT designed to assess the effects of exercise on fatigue; (2) It included all 456 patients receiving neurotoxic chemotherapy regimens (taxane-, platinum-, or vinca alkaloid-based chemotherapy) from the RCT. From the 420 patients who completed baseline assessments, 355 patients (85%) also completed post-intervention assessments (170 exercisers, 185 controls); (3) Patients reported CIPN symptoms of numbness and tingling and hot/coldness in hands/feet (0–10 scales) pre- and post-intervention. It was explored baseline neuropathy, sex, age, body mass index, cancer stage, and cancer type as possible factors associated with CIPN symptoms and exercise effectiveness	(1) Exercise reduced CIPN symptoms of hot/coldness in hands/feet and numbness and tingling compared to the control; (2) Exercise reduced CIPN symptoms more for patients who were older	(1) “Our results are consistent with cross-sectional evidence that more physical activity and larger muscle volume is associated with less severe CIPN symptoms”; (2) “Our results suggesting that exercise treats CIPN better for older patients are consistent with results that older patients require less exercise to treat CIPN”; (3) “Exercise shows promise in the treatment of CIPN and so this research should be continued, especially given the dearth of available treatments for CIPN”
[95]	Supervised SMT or WBV sessions twice a week, each lasting approximately 15 to 30 min, concomitant to medical therapy	(1) Prospective multicenter randomized clinical trial with 158 patients with cancer receiving chemotherapy (oxaliplatin or vinca alkaloids); (2) Patients were assigned to SMT, WBV, or treatment as usual in a 1:1:1 ratio; (3) All patients were assessed at baseline prior to initial chemotherapy and reassessed after 12 weeks + follow up 12 weeks after completion of chemotherapy	(1) The incidence of CIPN was significantly different across groups (treatment as usual: 70.6%, 95% CI, 58.0–83.2%; WBV: 41.2%, 95% CI, 27.9–54.5%; SMT: 30.0%, 95% CI, 17.9–42.1%); (2) SMT can decrease CIPN, maintain/improve vibration sensitivity, sense of touch, lower leg strength, pain, burning sensation, and balance control; patients needed fewer dose reductions and had less mortality, better quality of life, and higher physical activity levels; (3) WBV reduced incidence of CIPN and improved balance in a bipedal stance; (4) Patients receiving vinca alkaloids benefited most from SMT and WBV interventions, showing the lowest incidence of CIPN	(1) “The human neuromuscular system, if exposed to regular use and trained at maximum progression, seems to be able to maintain relevant neural functions even throughout chemotherapy”; (2) “Peripheral nerve regeneration is possible, and exercise plays a decisive role in maintaining and restoring neuromuscular function”
[96]	12-week EXCAP intervention (home-based, individually tailored, moderate-intensity walking and resistance exercise program)	(1) 90 patients (65 ± 11 years old, 52% women; cancer type: breast, gastrointestinal, multiple myeloma) starting neurotoxic chemotherapy were randomized to 12 weeks of exercise or active control (nutrition education). CIPN symptoms were assessed pre-, mid-, and post-intervention; (2) At pre- and post-intervention, it was used task-free (“resting”) fMRI to assess functional connectivity in the interoceptive brain system, involving the salience and default mode networks	(1) Moderate/large beneficial effects of exercise on CIPN symptoms, CIPN signs and physical function were observed; (2) Patients with worse CIPN had lower functional connectivity within the default mode network and higher functional connectivity within the salience network; (3) Exercise tended to increase hypoconnectivity and decrease hyperconnectivity seen in CIPN	(1) “Our small data set also tentatively suggests that exercise during neurotoxic chemotherapy can partially protect against CIPN with clinically meaningful benefits and that the interoceptive brain system plays a role in CIPN and its treatment by exercise”

EORTC QLQ C-30/LC-13: European Organization for Research and Treatment of Cancer Quality of Life Questionnaire Core-30; mCRC: metastasized colorectal cancer; EXCAP: Exercise for Cancer Patients © ^®^; SMT: sensorimotor training; WBV: whole-body vibration training; CI: confidence interval; fMRI: functional magnetic resonance imaging.

**Table 2:** Multidimensional impact of physical exercise in CIPN and underlying mechanisms.

Mechanism	Effect of Exercise	Impact on CIPN	References
**(1)** **Neurotrophic factors**	Increased expression of GDNF, BDNF and IGF-1	Promotes axonal regeneration, cellular survival, and neuroprotection	[14,99,100]
**(2)** **Anti-inflammation and antioxidation**	Release of anti-inflammatory cytokines (IL-10 and IL-1RA (…)); reduction of oxidative stress markers	Ameliorates CIPN via anti-inflammatory cascades	[14]
**(3)** **Opioid system**	Increases activation of MOR, KOR, and DOR by endorphins, enkephalins and dynorphins	Analgesia; pain modulation	[103,104,105]
**(4)** **Monoaminergic system**	Activates noradrenergic system; increases 5-HT in pain control areas of the brain	Pain modulation	[112,116,117,118]
**(5)** **Endocannabinoid system**	Increases endocannabinoid levels; activates CB1 and CB2 receptors	Analgesia, decreases mechanical allodynia and thermal hyperalgesia, euphoria	[122,124,125,126,127,128,129]
**(6)** **Human brain networks**	Increases connectivity in important neurocircuits (salience network (including the amygdala and the anterior cingulate cortex), executive control network, and default mode network (including the hippocampus))	Psychological well-being	[135,136,137,138,139,140,141]
**(7)** **Psychological well-being**	Release of endorphins, neurotrophic factors and neurotransmitters, involvement of the hypothalamic-pituitary-adrenal axis reducing stress and improving mood	Psychological well-being	[142]
**(8)** **Gut microbiome**	Influences gut microbiota composition and diversity	Decreased inflammatory signaling, increased neuroprotection; antidepressant effects	[143,144,145,146,153,154]

## Data Availability

Not applicable.

## Abbreviations

The following abbreviations are used in this manuscript:

CIPN	Chemotherapy-induced peripheral neuropathy
RCT	Randomized controlled trial
SNPs	Single-nucleotide polymorphisms
CNS	Central nervous system
PNS	Peripheral nervous system
TRP	Transient receptor potential
DRG	Dorsal root ganglion
GABA	Gamma-aminobutyric acid
GPCR/MAPK	G protein-coupled receptor/mitogen-activated protein kinase
PAG	Periaqueductal gray matter
IL	Interleukin
TNF-alpha	Tumor necrosis factor alpha
5-HT/NA	Serotonin and noradrenaline
RVM	Rostral ventromedial medulla
EORTC QLQ C-30/LC-13	European Organization for Research and Treatment of Cancer Quality of Life Questionnaire Core-30
mCRC	Metastasized colorectal cancer
EXCAP	Exercise for Cancer Patients © ^®^
SMT	Sensorimotor training
WBV	Whole-body vibration training
CI	Confidence interval
fMRI	Functional magnetic resonance imaging
GDNF	Glial cell line-derived neurotrophic factor
BDNF	Brain-derived neurotrophic factor
IGF-1	Insulin-like growth factor
MOR	Mu opioid receptor
DOR	Delta opioid receptor
KOR	Kappa opioid receptor
CB1	Cannabinoid receptor type 1
CB2	Cannabinoid receptor type 2
NO	Nitric oxide
SCFAs	Short-chain fatty acids
